# Genome-wide identification of LRR-containing sequences and the response of these sequences to nematode infection in *Arachis duranensis*

**DOI:** 10.1186/s12870-018-1508-x

**Published:** 2018-11-13

**Authors:** Hui Song, Zhonglong Guo, Tao Chen, Juan Sun, Guofeng Yang

**Affiliations:** 1 Grassland Agri-husbandry Research Center, Qingdao Agricultural University, Qingdao, 266109 China; 2 State Key Laboratory of Protein and Plant Gene Research, Peking-Tsinghua Center for Life Sciences, School of Life Sciences and School of Advanced Agricultural Sciences, Peking University, Beijing, 100871 China; 3 State Key Laboratory of Grassland Agro-ecosystems, College of Pastoral Agriculture Science and Technology, Lanzhou University, Lanzhou, 730000 China

**Keywords:** Co-expression, LRR-containing gene, Nematode, Phylogenetic relationship, Substitution rate

## Abstract

**Background:**

Leucine-rich repeat (LRR)-containing genes are involved in responses to various diseases. Recently, RNA-seq data from *A. duranensis* after nematode (*Meloidogyne arenaria*) infection were released. However, the number of LRR-containing genes present in *A. duranensis* and the response of LRR-containing genes to nematode infection are poorly understood.

**Results:**

In this study, we found 509 amino acid sequences containing nine types of LRR domains in *A. duranensis*. The inferred phylogenetic relationships revealed that the nine types of LRR domains had two originations. The inferred selective pressure was mainly consistent with LRR domains undergoing purifying selection. Twenty-one LRR-containing genes were associated with possible resistance to nematode infection after 3, 6, and 9 days. Among them, Aradu.T5WNW, Aradu.JM17V, and Aradu.MKP1A were up-regulate at these three time points, while Aradu.QD5DS and Aradu.M0ENQ were up-regulated 6 and 9 days after nematode infection. The expression of the above mentioned five genes was significantly and negatively correlated with the number of LRR8 domain, indicating that fewer LRR8 domains are associated with the promotion of LRR-containing genes that resist nematode infection. Patterns of co-expression and *cis*-acting elements indicated that WRKY possibly regulate the responses of LRR-containing genes to nematode infection and that expansin genes may work together with LRR-containing genes in response to nematode infection.

**Conclusions:**

We identified the number and type of LRR-containing genes in *A. duranensis*. The LRR-containing genes that were found appear to be involved in responses to nematode infection. The number of LRR8 domains was negatively correlated with expression after nematode infection. The WRKY transcription factor may regulate resistance to nematode infection based on LRR-containing genes. Our results could improve the understanding of resistance to nematodes and molecular breeding in peanuts.

**Electronic supplementary material:**

The online version of this article (10.1186/s12870-018-1508-x) contains supplementary material, which is available to authorized users.

## Background

Cultivated peanut (*Arachis hypogaea*) is a major oil and protein crop worldwide. Cultivated peanut is an allopolyploid hybrid between the wild diploid species *Arachis duranensis* and *A*. *ipaënsis* [1, 2]. To date, the genome sequences of *A*. *duranensis* and *A*. *ipaënsis* have been sequenced and released [1]. Biotic and abiotic stresses are among the main reasons for production losses in peanut crops. Previous studies have shown that resistance to biotic stress in wild peanut is stronger than that in cultivated peanut [3–6]. Accordingly, it is important to research the evolutionary and expression patterns of gene families and specific genes in wild peanut because such studies may provide resources for improving the biotic stress resistance of cultivated peanut. Currently, the genome-wide identification and characterization of basic/helix-loop-helix (bHLH), expansin (EXP), heat shock transcription factor (HSF), lipoxygenase (LOX), nucleotide binding–leucine rich repeat (NBS–LRR), and WRKY gene families in wild peanut have been reported [7–12]. The expression profiles of NBS–LRR between *A. duranensis* and *A. hypogaea* revealed high expression in *A. duranensis* but not in *A. hypogaea* under *Aspergillus flavus* treatment [7].

Leucine-rich repeat (LRR) proteins typically contain repeats composed of 20–29 residues, such that they constitute a continuous parallel β-sheet, which forms helically twisted and solenoid-like structures [13]. LRR-containing proteins play crucial roles in protein–ligand and protein–protein interactions; these LRR-containing proteins are involved in plant immune responses and mammalian innate immune responses [13, 14]. In our previous study, we found that the LRR5 domain only appeared in CNL sequences and that only LRR8 domains of paralogs underwent positive selection in *A*. *duranensis* and *A*. *ipaënsis* [7]. However, little is known about which genes contain LRR domains, what selective pressures have acted on these domains throughout their evolution, and which LRR-containing genes are involved in biotic stress responses in *Arachis*. Previous field and QTL studies have shown that the effect of biotic stress on *A*. *duranensis* was significantly stronger than that on *A*. *ipaënsis* [6, 15]. The RNA-seq datasets from *A*. *duranensis* after nematode infection have previously been completed and released [16]. In this study, we first identified LRR-containing genes using a bioinformatic approach in *A*. *duranensis.* Next, we analyzed the substitution rates of these LRR-containing paralogs and evaluated which LRR-containing genes respond to nematode infection. Finally, we determined which transcription factors regulate LRR-containing genes and which genes work together with LRR-containing genes in responding to nematode infections. These results could provide the basis for future evolutionary biological studies of LRR-containing genes and their resistance to nematode infection.

## Methods

### Identification of sequences with LRR domains

A total of 17 hidden Markov models (HMMs) of LRR domains were documented in the Pfam public database (http://pfam.xfam.org/). These HMM models include LRR19-TM (PF15176), LRRC37 (PF15779), LRRC37AB_C (PF14914), LRRCT (PF01463), LRRFIP (PF09738), LRRNT (PF01462), LRRNT_2 (PF08263), LRR_1 (PF00560), LRR_2 (PF07723), LRR_3 (PF07725), LRR_4 (PF12799), LRR_5 (PF13306), LRR_6 (PF13516), LRR_8 (PF13855), LRR_9 (PF14580), LRR_adjacent (PF08191), and LRV (PF01816). The complete *A. duranensis* genome has been sequenced [1], and the annotated sequences were downloaded from the PeanutBase website (https://peanutbase.org/files/genomes/Arachis_duranensis/). The 17 HMMs were downloaded and LRR-containing sequences were detected using the HMMER program [17] and *A. duranensis* amino acid sequences. The LRR-containing sequences were extracted using an in-house Perl script. To exclude the false-positive sequences, all sequences were uploaded to the Pfam public database to verify LRR domains.

### Phylogenetic relationship

Multiple sequence alignment of full-length amino acid and LRR domain sequences was conducted using MAFFT 7.0 [18]. Phylogenetic trees were then constructed using MEGA 6.0 [19] according to maximum likelihood with the Jones–Taylor–Thornton (JTT) model based on 1000 bootstrap replicates. Gene sequences that clustered in the phylogenetic tree with a bootstrap value greater than 70 were considered paralogous [9]. The PAL2NAL program [20] was used for the conversion of amino acid sequences into the corresponding nucleotide sequences. PAML 4.0 [21] was used to calculate the nonsynonymous to synonymous substitution ratio (*K*_a_/*K*_s_). Generally, *K*_a_/*K*_s_ values of 1, > 1, and < 1 indicate neutral, positive, and purifying selection, respectively.

### The expression of LRR-containing sequences in different tissues and under nematode infection

RNA-seq datasets from 22 different *A. duranensis* tissues have been released on the PeanutBase website (https://peanutbase.org/gene_expression/atlas) [22, 23]. The genes differentially expressed in root tissue after 3, 6, and 9 days of nematode infection have been published on PeanutBase as well (https://peanutbase.org/gene_expression/atlas_nematode). The sequencing details were described in previous publications [16, 23]. In this study, the heat maps were generated in R using the heatmap.2 function available in the gplots CRAN library package. The fragments per kilobase of transcript per million mapped reads (FPKM) value for each gene was normalized using a log_2_-transformation.

### Co-expression analyses

Genes co-expressed under nematode infection were detected among 22 different tissues using a weighted gene co-expression network analysis (WGCNA) script in R [24]. Differentially expressed genes with a log_2_-fold change of greater than 2 or less than − 2 were used for WGCNA analyses. A soft threshold (*β*) value of 12 was used in the transformation of the adjacency matrix in order to meet the scale-free topology criteria. Co-expression modules were created with the blockwiseModules function using the following parameters: power = 6, TOMType = “unsigned,” maxBlockSize = 30,000, mergeCutHeight = 0.25, minModuleSize = 30, reassignThreshold = 0.

Gene ontology (GO) annotations for genes in each module containing the expansin gene were extracted from the *A. duranensis* genome available on the PeanutBase website (https://peanutbase.org/files/genomes/Arachis_duranensis/) [22].

### Identification of *cis*-acting element

The 2 kb *cis*-acting element sequence of the full-length LRR-containing gene was retrieved from the PeanutBase website (https://peanutbase.org/genomes/jbrowse/?data=Aradu1.0). Transcription regulatory elements were predicted using NSITE [25] with a *P*-value threshold of 0.05, and transcription factor annotation was retrieved from the PlantTFDB 4.0 database [26].

## Results

### LRR domains in *A. duranensis*

In this study, a total of nine types of LRR domains associated with 1403 sequences were identified in *A. duranensis*. These LRR domains included LRRNT_2 (316 sequences), LRR_1 (221 sequences), LRR_2 (10 sequences), LRR_3 (33 sequences), LRR_4 (22 sequences), LRR_5 (1 sequences), LRR_6 (155 sequences), LRR_8 (643 sequences), and LRR_9 (2 sequences; Table 1 and Additional file 1: Table S1). These nine LRR domains were randomly distributed on ten chromosomes. Among them, LRR1, LRR6, LRR8, and LRRNT_2 domains were detected on all chromosomes (Table 1). However, there were more LRR8 domains than all other domains on each chromosome (Table 1).

**Table 1:** The number of LRR domains in *Arachis duranensis* by chromosome

Domain	Chr A1	Chr A2	Chr A3	Chr A4	Chr A5	Chr A6	Chr A7	Chr A8	Chr A9	Chr A10	Scaffold	Total
LRR1	16	24	22	34	27	14	37	11	14	22	0	221
LRR2	0	0	0	2	0	0	0	0	7	1	0	10
LRR3	0	4	6	1	0	1	0	4	17	0	0	33
LRR4	1	0	3	3	3	2	2	1	2	5	0	22
LRR5	0	0	0	1	0	0	0	0	0	0	0	1
LRR6	15	26	6	18	16	9	30	6	22	6	1	155
LRR8	43	62	54	115	59	41	130	38	35	64	2	643
LRR9	0	0	0	1	1	0	0	0	0	0	0	2
LRRNT_2	20	22	35	67	35	18	47	14	25	33	0	316

Note: Chr indicates chromosome

The 1403 LRR sequences were distributed among 509 amino acid sequences in *A. duranensis*. Among them, 317, 49, 48, 36, 30, 11, and 18 sequences belonged to the receptor-like kinase, NBS–LRR, protein kinase, LRR receptor, F-box, ATP binding, and other gene families, respectively (Additional file 1: Table S1). Further, the LRR3 domain was only detected in the NBS–LRR gene family, and other domains were at least found in two different gene families (Additional file 1: Table S1). In addition, one amino acid sequence contained up to five domain types and contained the most LRR sequences, up to 21 (Additional file 1: Table S1).

### Phylogenetic analyses

**Fig. 1:**
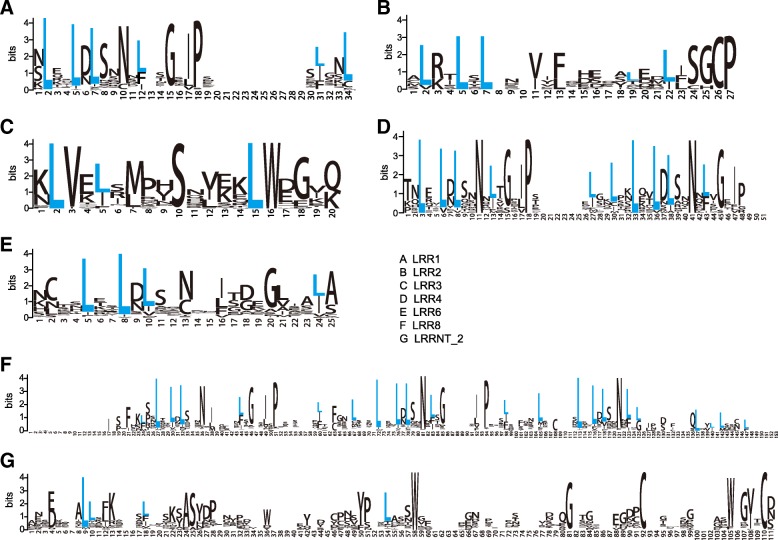
The different LRR domains in *Arachis duranensis.* Each blue letter “L” indicates a leucine amino acid

The multiple alignment results showed that structures differed between LRRNT_2 and other LRR domains. Among LRR1, 2, 3, 4, 5, 6, 8, and 9 domains, the LRR domain was composed of a typical LxxLxLxx repeat unit (where L is leucine and x is any other amino acid), but this feature was absent from the LRRNT_2 domain (Fig. 1). The results indicated that LRR1, 2, 3, 4, 5, 6, 8, and 9 domains have a similar biological function and a common origin.

**Fig. 2:**
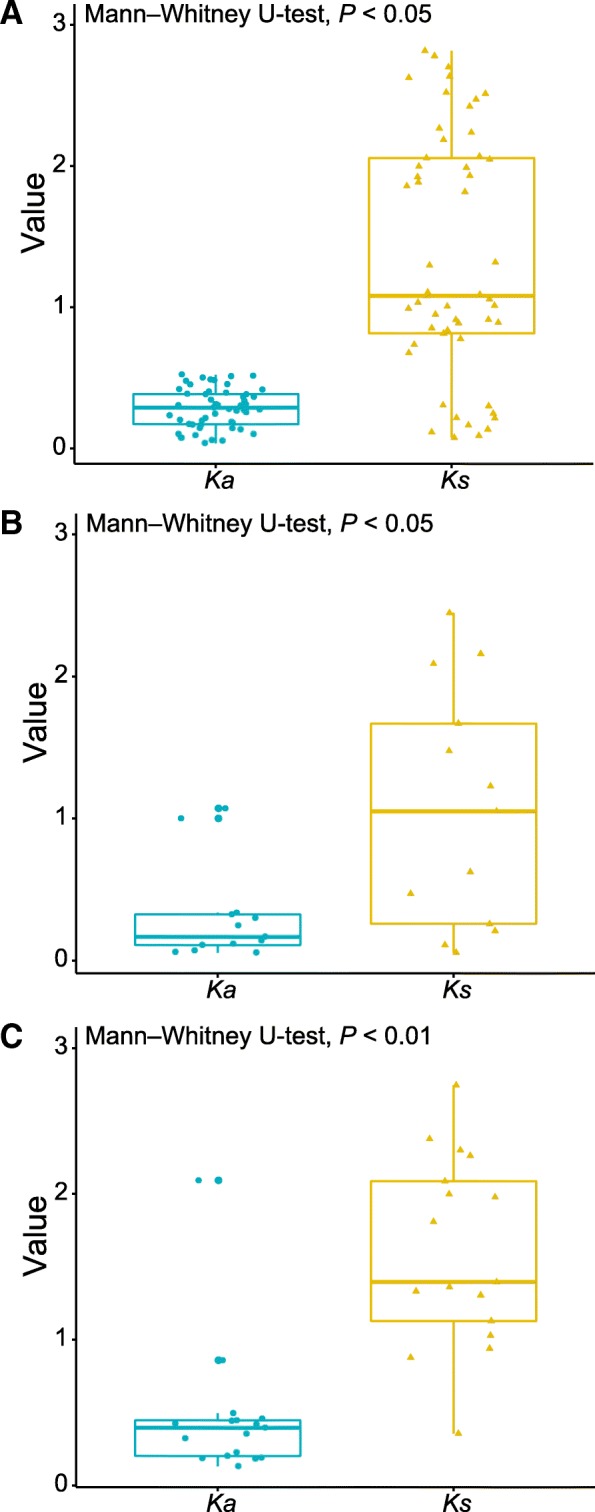
The substitution rate in LRR-containing paralogs .**a** *K*_a_ versus *K*_s_ in LRR-containing paralogs; **b** *K*_a_ versus *K*_s_ in LRR8 domains; **c** *K*_a_ versus *K*_s_ in LRRNT_2 domains

To ensure accurate inference of the topological structures, we used a computationally efficient maximum likelihood method to construct phylogenetic trees. The phylogenetic tree indicated that 1, 4, 3, 9, 71, and 3 paralogous genes were detected among ATP binding, F-box, LRR receptor, NBS–LRR, protein kinase, and receptor-like kinase genes, respectively (Additional file 2: Figure S1, Additional file 3: Figure S2, Additional file 4: Figure S3, Additional file 5: Figure S4, Additional file 6: Figure S5 and Additional file 7: Figure S6). The number of protein kinase genes is higher because protein kinases have underwent more duplication or been retained at higher rates after gene duplication events [27]. The substitution rate results have shown that the average synonymous substitution rate (*K*_s_ value; 1.36) of these paralogs was significantly higher than the average nonsynonymous substitution rate (*K*_a_ value; 0.29, Mann–Whitney U-test, *P* < 0.01, Fig. 2a). The average nonsynonymous to synonymous substitution ratio (*K*_a_/*K*_s_ value) was 0.28. These results indicated that purifying selection has acted on these paralogs. Further, the average *K*_a_/*K*_s_ value of LRR domains were less than 1 except for three pairs of domains (LRR6, LRR8, and LRRNT_2). The average *K*_s_ value of LRR domains exceeded the average *K*_a_ value. Among them, LRR8 (Mann–Whitney U-test, *P* < 0.05, Fig. 2b) and LRRNT_2 (Mann–Whitney U-test, *P* < 0.01, Fig. 2c) exhibited statistically significant differences between *K*_s_ and *K*_a_ values. These results indicated the LRR domain mainly underwent purifying selection.

### The expression of LRR-containing genes among 22 different tissues

**Fig. 3:**
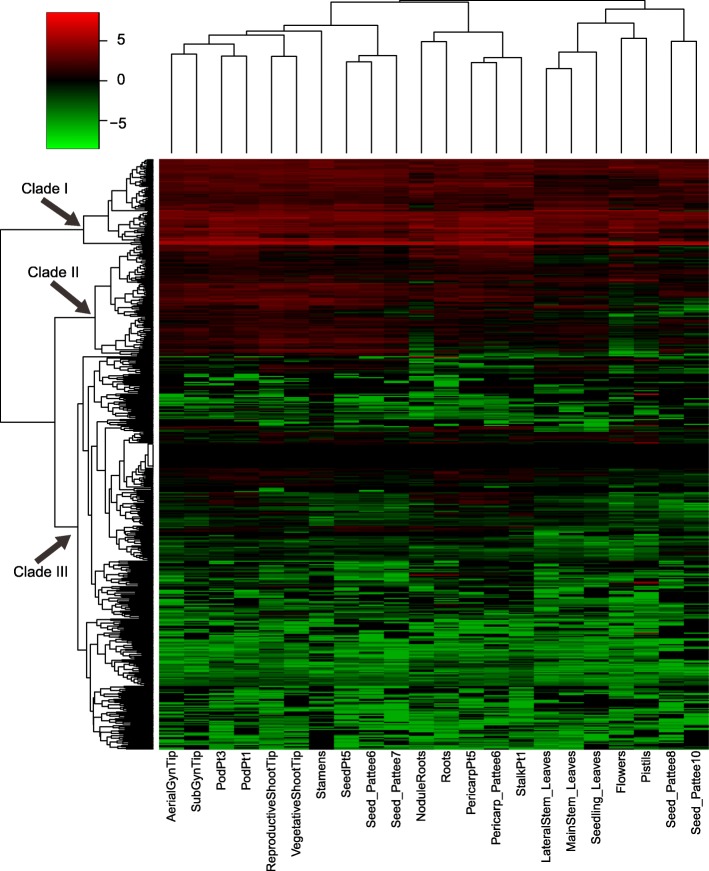
The expression of LRR-containing genes in 22 different *Arachis duranensis* tissues. Seedling_Leaves, seedling leaf 10 d post emergence; MainStem_Leaves, main stem leaf; LateralStem_Leaves, lateral leaf; VegetativeShootTip, vegetative shoot tip from the main stem; ReproductiveShootTip, reproductive shoot tip from the first lateral leaf; Roots, 10 d roots; NoduleRoots, 25 d nodules; Flowers, perianth; Pistils, gynoecium; Stamens, androecium; AerialGynTip, aerial gynophore tip; SubGynTip, subterranean gynophore tip; PodPt1, Pattee stage 1 pod; StalkPt1, Pattee stage 1 stalk; PodPt3, Pattee stage 3 pod; Pericarp_Pattee5, Pattee stage 5 pericarp; Seed_Pattee5, Pattee stage 5 seed; Pericarp_Pattee6, Pattee stage 6 pericarp; Seed_Pattee6, Pattee stage 6 seed; Seed_Pattee7, Pattee stage 7 seed; Seed_Pattee8, Pattee stage 8 seed; Seed_Pattee10, Pattee stage 10 seed

The 509 LRR-containing genes can be classified into three groups based on their expression among 22 different tissues, including clades I, II, and III (Fig. 3). These 74 genes in clade I and 339 genes in clade III have high and low expression in 22 different tissues, respectively. Ninety-six genes in clade II showed moderate expression. In clade II, these genes were expressed highly in aerial gynophore tip, subterranean gynophore tip, Pattee stage 1 pod, Pattee stage 3 pod, vegetative shoot tip (from the main stem), reproductive shoot tip (from the first lateral leaf), androecium, Pattee stage 5 seed, Pattee stage 6 seed, and Pattee stage 7 seed tissues. However, low gene expression was exhibited in seedling leaf (10 d post-emergence), main stem leaf, lateral leaf, perianth, gynoecium, Pattee stage 8 seed, and Pattee stage 10 seed tissues (Fig. 3). Notable, NBS–LRR genes were mainly distributed in clade III, and NBS–LRR genes were absent from clade I among all 22 different tissues. The results showed that NBS–LRR genes have constitutive but low expression.

### The response of LRR-containing genes to nematode infection

**Fig. 4:**
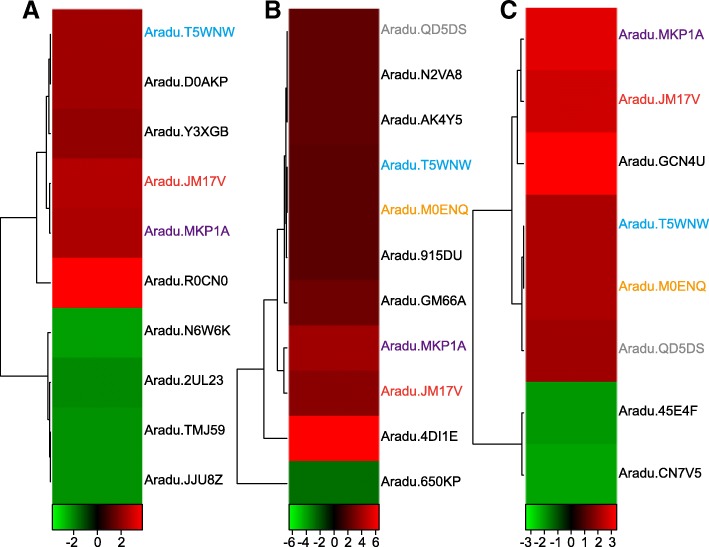
The differentially expressed LRR-containing genes after nematode infection. **a** The differential expression of LRR-containing genes 3 days after nematode infection. **b** The differential expression of LRR-containing genes 6 days after nematode infection. **c** The differential expression of LRR-containing genes 9 days after nematode infection

**Fig. 5:**
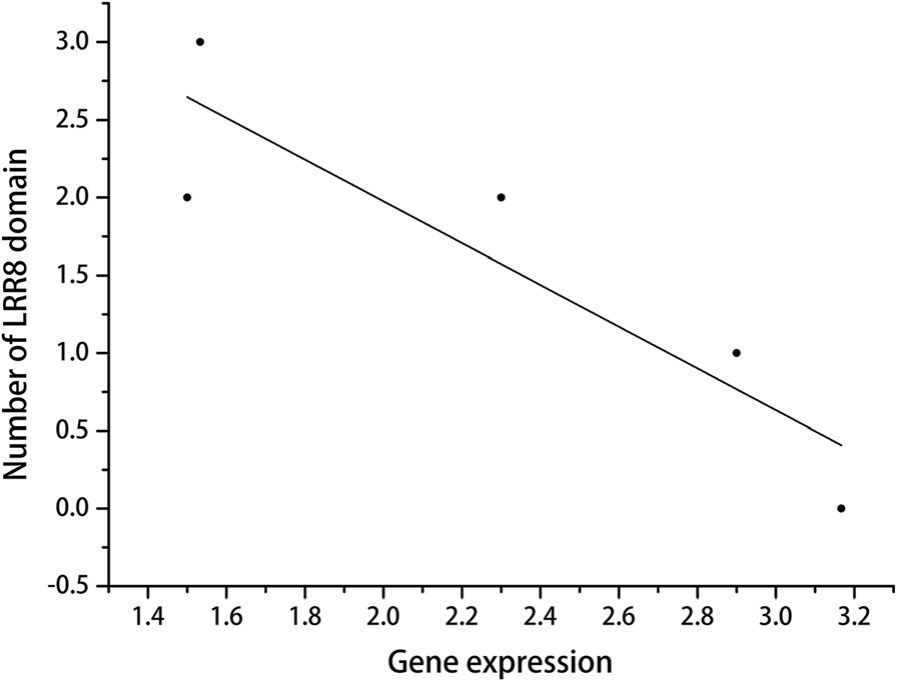
The correlation between the number of LRR8 domains and gene expression involved in resistance to nematode infection

The RNA-seq results revealed that 21 LRR-containing genes were differentially expressed in root tissues under nematode infection after 3, 6, and 9 days (Fig. 4 and Additional file 8: Table S2), indicating these genes are possibly involved in resistance to nematode infection. Among them, the expression levels of Aradu.T5WNW (LRR8-containing gene, receptor-like kinase), Aradu.JM17V (LRR1- and LRR8-containing gene, disease resistance protein), and Aradu.MKP1A (LRR3-containing gene, NBS–LRR) were up-regulate at three time points, and those of Aradu.QD5DS (LRRNT_2- and LRR8-containing gene, LRR receptor) and Aradu.M0ENQ (LRRNT_2-, LRR1-, LRR6-, and LRR8-containing gene, receptor-like kinase) were up-regulated under nematode infection after 6 and 9 days. However, no genes exhibited down-regulated expression at more than one time point. These results indicated that the above mentioned five genes are possibly involved in resistance to nematode infection. The correlation analysis found that expression of the above mentioned five genes was significantly and negatively correlated with the number of LRR8 domains (*r* = − 0.9, *P* < 0.05, Fig. 5) and exhibited a non-significant negative correlation with the number of other domains. These results suggested that fewer LRR8 domains are associated with promoting resistance to nematode infection via LRR-containing genes.

**Fig. 6:**
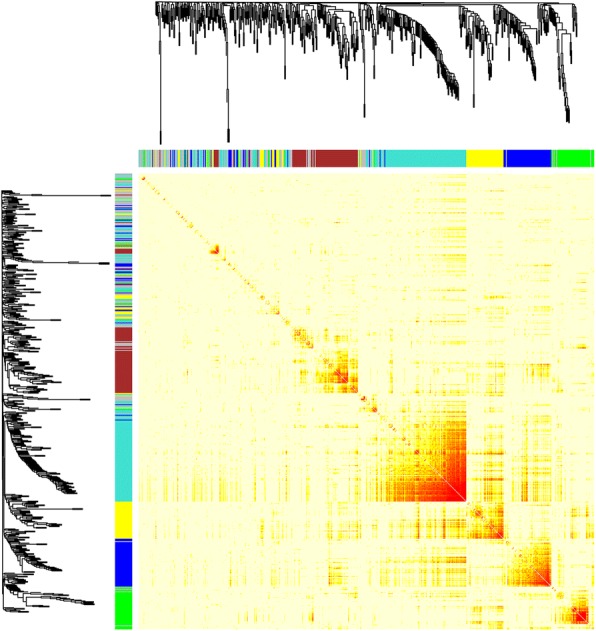
The expression of LRR-containing and co-expressed genes in 22 different tissues. Branches in the hierarchical clustering dendrograms correspond to modules. Color-coded module membership is displayed in the colored bars below and to the right of the dendrograms. High co-expression interconnectedness is indicated by progressively more saturated yellow and red coloration

**Fig. 7:**
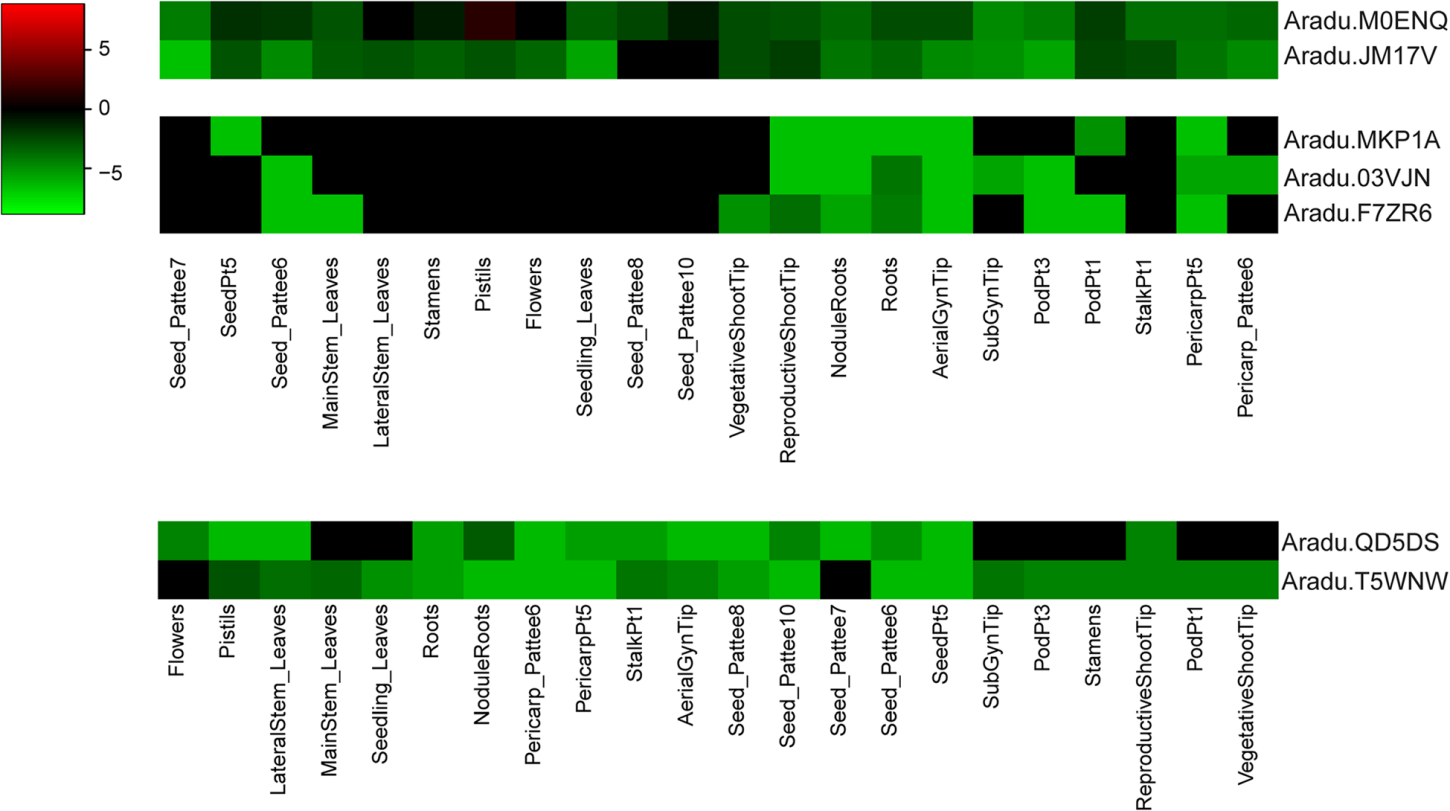
The similar expression patterns between LRR-containing genes and other genes co-expressed during resistance to nematode infection. Seedling_Leaves, seedling leaf 10 d post emergence; MainStem_Leaves, main stem leaf; LateralStem_Leaves, lateral leaf; VegetativeShootTip, vegetative shoot tip from the main stem; ReproductiveShootTip, reproductive shoot tip from the first lateral leaf; Roots, 10 d roots; NoduleRoots, 25 d nodules; Flowers, perianth; Pistils, gynoecium; Stamens, androecium; AerialGynTip, aerial gynophore tip; SubGynTip, subterranean gynophore tip; PodPt1, Pattee stage 1 pod; StalkPt1, Pattee stage 1 stalk; PodPt3, Pattee stage 3 pod; Pericarp_Pattee5, Pattee stage 5 pericarp; Seed_Pattee5, Pattee stage 5 seed; Pericarp_Pattee6, Pattee stage 6 pericarp; Seed_Pattee6, Pattee stage 6 seed; Seed_Pattee7, Pattee stage 7 seed; Seed_Pattee8, Pattee stage 8 seed; Seed_Pattee10, Pattee stage 10 seed

A total of 462 genes were used to classify five modules after nematode infection in co-expression analyses (Fig. 6). Aradu.JM17V, Aradu.M0ENQ, and Aradu.MKP1A genes were distributed in module 1, and Aradu.QD5DS and Aradu.T5WNW genes were found in module 3. These results showed that although these five genes involved in nematode infection resistance, Aradu.JM17V, Aradu.M0ENQ, and Aradu.MKP1A have similar expression patterns, and Aradu.QD5DS and Aradu.T5WNW have similar expression patterns across 22 different tissues. In modules 1 and 3, we found some transcription factors (such as WRKY, bHLH, and Trihelix) that were involved in responding to insects or biotic stress (Table 2). Analysis of the *cis*-acting elements showed that the sequences upstream of these LRR-containing genes can bind some transcription factors (Aradu.T5WNW was excluded from this analysis because it is a partial sequence.). These transcription factor binding sites included Dof, BP, bHLH, RTCS, BZZR1, Hox1a, E2F2, WRKY, GT-1, Hsf, and AGL15 (Table 3). These results indicated the WRKY transcription factor may regulate Aradu.M0ENQ, while WRKY and/or bHLH transcription factors may regulate the response of Aradu.MKP1A to nematode infection. In addition, we found that some genes have potential roles in resisting nematode infection or biotic stress responses (Table 4) based on previous studies [9, 10]. For example, the overexpression of the expansin-like B gene from *A. hypogaea* in transformed soybean plants remarkably decreased the number of galls in transformed hairy roots inoculated with nematodes [10]. Our results indicated the above mentioned five proteins possibly interacted with these genes, including expansin and lipoxygenase genes. Further, two gene pairs (Aradu.M0ENQ and Aradu.JM17V; Aradu.QD5DS and Aradu.T5WNW) had similar expression patterns, respectively (Fig. 7). The expression pattern of Aradu.MKP1A was similar to those of Aradu.03VJN and Aradu.F7ZR6 (Fig. 7). These results indicated these genes may have synergistic effects on resistance to nematode infection.

**Table 2:** The transcription factors associated with resistance to biotic stress in modules 1 and 3

Gene ID	Gene Name	Go annotation
Module 1		
Aradu.B7RDX	Basic helix-loop-helix (bHLH) DNA-binding factor	GO:0046983 (protein dimerization activity)
Aradu.C8ZMR	WRKY family transcription factor	GO:0003700 (sequence-specific DNA binding transcription factor activity), GO:0043565 (sequence-specific DNA binding)
Aradu.CAP3X	WRKY family transcription factor	GO:0003700 (sequence-specific DNA binding transcription factor activity), GO:0043565 (sequence-specific DNA binding)
Aradu.KG41H	WRKY family transcription factor	GO:0003700 (sequence-specific DNA binding transcription factor activity), GO:0043565 (sequence-specific DNA binding)
Aradu.S0KU9	Basic helix-loop-helix (bHLH) DNA-binding factor	GO:0046983 (protein dimerization activity)
Aradu.S7YD6	WRKY family transcription factor	GO:0003700 (sequence-specific DNA binding transcription factor activity), GO:0043565 (sequence-specific DNA binding)
Aradu.V6U4I	WRKY family transcription factor	GO:0003700 (sequence-specific DNA binding transcription factor activity), GO:0043565 (sequence-specific DNA binding)
Module 3		
Aradu.752ZV	Basic helix-loop-helix (bHLH) DNA-binding factor	GO:0003677 (DNA binding), GO:0046983 (protein dimerization activity)
Aradu.B6WSD	Trihelix transcription factor	GO:0003682 (chromatin binding)

**Table 3:** Bound transcription factor sites in LRR-containing genes associated with resistance to nematode infection

Gene ID	Binding transcription factor
Aradu.JM17V	DOF1 (1,Dof family); BP (2,BBR-BPC family);RTCS(2,LBD family);BZR1 (1,BES1 family)
Aradu.M0ENQ	DOF1 (1,Dof family); BP (1,BBR-BPC family); Hox1a(1,HD-ZIP family);WRKY11 (2,WRKY family); E2F2 (1,E2F/DP family)
Aradu.MKP1A	GT-1 (1,Trihelix family); BP (1,BBR-BPC family); WRKY1 (1,WRKY family); Hsf (1,bHLH family); Hsf (1,HSF family)
Aradu.QD5DS	AGL15 (1,MADS family)
Aradu.T5WNW	No detection

**Table 4:** The genes inferred to interact with LRR-containing genes involved in resistance to nematode infection

Gene ID	Gene Name	GO annotation
Module 1		
Aradu.0QC7R	Expansin-like B1	GO:0005576 (extracellular region)
Aradu.4N3HV	Disease resistance family protein;	GO:0006952 (defense response), GO:0043531 (ADP binding)
Aradu.8D3SW	Lipoxygenase 3	No detection
Aradu.G29LA	Disease resistance protein	GO:0005515 (protein binding), GO:0006952 (defense response), GO:0007165 (signal transduction), GO:0043531 (ADP binding)
Aradu.J8T8R	Disease resistance protein	GO:0043531 (ADP binding)
Aradu.KH5IZ	Expansin-like B1	GO:0005576 (extracellular region)
Aradu.YUN33	Expansin-like B1	No detection
Module 3		
Aradu.A0K1D	MLP-like protein	GO:0006952 (defense response), GO:0009607 (response to biotic stimulus)

## Discussion

LRR domains were distributed among many proteins [14]. LRR domains have co-evolved with pathogen effectors, and their roles have been recognized directly or indirectly through pathogen effects [28]. In this study, 509 LRR-containing genes, including receptor-like kinase, NBS–LRR, protein kinase, LRR receptor, F-box, and ATP binding gene families, were detected in *A. duranensis*. *A. duranensis* LRR-containing genes were less numerous than those of *Arabidopsis thaliana* (700) and *Oryza sativa* subsp. *japonica* (1400) [29]. However, we found different types of LRR domains were biased among different gene families in *A. duranensis*. For example, the LRR3 domain was mainly found in the NBS–LRR gene family.

The LRR domain has two features that were identified by previous studies. First, the LRR domain contains a conserved consensus sequence LxxLxLxx (where x is any amino acid and L is leucine) [13]. Second, most paralogs that are involved in defense functions have underwent positive selection as their *K*_a_/*K*_s_ values exceeded 1 [30–32]. In this study, however, we found that most paralogs were subjected to purifying selection. There are at least two explanations for these apparent differences. First, we distinguished the different types of LRR domains before estimating substitution rates in this study, but substitution rates were calculated among all types of LRR domains in previous studies. Accordingly, false positives may have possibly been decreased by our choice to analyze the different types of LRR domains individually. Indeed, approximately 50% of automatically reported instances of positive selection have been revealed to be false positives after manual curation in a previous study [31]. Second, the various substitution rates were distributed in LRR-containing genes between *A. duranensis* and other plants.

To reveal the origin of LRR domains, we constructed a phylogenetic tree using all 1403 LRR domains; however, no adequately common sites were identified for construction of a phylogenetic tree (data not shown). However, the multiple alignment results indicated that the nine types of LRR domains form at least two groups (consistent with multiple origins). LRR1, 2, 3, 4, 5, 6, 8, and 9 domains contained the LxxLxL repeat unit, but LRRNT_2 contained fewer L amino acids. Nevertheless, we attempted to estimate whether LRR1, 2, 3, 4, 5, 6, 8, and 9 have an ancient ancestor. Previous studies have disputed the origin of LRR domains. Kajava [33] suggested separate origins for several different types of LRR domains based on the high levels of conservation within each LRR class. In contrast, Andrade et al., [15] found that LRR domains have a common origin rather than separate origins because homology-based methods could not absolutely partition LRR domains into these separate classes [34].

Although some reports have demonstrated that receptor-like kinase, NBS–LRR, protein kinase, LRR receptor, and F-box gene families are involved in resistance to pathogen infection [13, 35–37], to the best of our knowledge, only NBS–LRR genes conferred resistance to nematode infection [16, 38, 39]. Peanut yield losses were affected by infections of fungi, bacteria, virus, and nematode (*Meloidogyne arenaria*) [40]. Peanuts infected with nematode present symptoms such as stunted growth, wilting, and enhanced susceptibility to other pathogens [41]. In this study, we found that NBS–LRR (Aradu.MKP1A), receptor-like kinase (Aradu.T5WNW and Aradu.M0ENQ), LRR receptor (Aradu.QD5DS), and disease resistance protein (Aradu.JM17V) genes were involved in responses to nematode infection. Therefore, more studies on these genes could clarify the gene regulatory networks that respond to nematode infections and help decrease nematode infections.

The genes with more LRR domains tend to be associated with resistance to biotic stress [42]. For example, the rice *Xa21* gene has 23 LRR copies and confers resistance to the bacterial blight pathogen *Xanthomonas oryzae* pv. *oryzae* race 6 [43], and *FLS2* in *Arabidopsis*, with 28 LRRs, was involved in flagellin response [44]. Unexpectedly, we found that the number of LRR domains was negatively correlated with gene expression after nematode infection, and in particular, the LRR8 domain was significantly and negatively correlated with gene expression in *A. duranensis*. There is at least one explanation for this difference. Pattern-triggered immunity (PTI), but not effector-triggered immunity (ETI), plays a crucial role in resistance to nematode infection. To date, the innate immunity system can be classified into two layers, PTI and ETI [45]. The former layer is mediated by surface-localized pattern recognition receptors (PRRs) that recognize pathogen-associated molecular patterns (PAMPs) of pathogens. The second layer, ETI, is involved in intracellular immune receptors, which directly or indirectly depend on resistance genes (*R* genes) and resistance to invasion of pathogens. The LRR domain in *R* genes have co-evolved with pathogen effectors, and their role was recognized directly or indirectly with pathogen molecules [28]. However, Manosalva et al. [46] found that PTI could be activated during nematode infection in monocots and dicots.

In this study, we found that WRKY transcription factors can regulate the response of LRR-containing genes to nematode infection. This finding was consistent with previous results. Sarris et al. [47] demonstrated that the bacterial effectors AvrRps4 or PopP2 can bind WRKY transcription factors that are involved in active NBS–LRR gene responses to pathogens. In peanut, Guimarães et al. [16] found that most WRKY genes function as transcription factors, playing a key role in this incompatible plant–nematode interaction and indicating that WRKY possibly regulates other gene responses to nematode infection. In addition, the RNA-seq data from *A. duranensis* showed that more expansin genes were up-regulated after nematode infection [16]. We found that three expansin genes (Aradu.0QC7R, Aradu.KH5IZ, and Aradu.YUN33) were detected in module 1, indicating that expansin genes could work together with LRR-containing genes in responding to nematode infections.

## Conclusions

We identified the number and type of LRR-containing genes in *A. duranensis*. We further estimated the substitution rate of each type of LRR domain between paralogs. LRR domains were inferred to have mainly been subject to purifying selection. In addition, we comprehensively identified the LRR-containing genes that were involved in responses to nematode infection. The number of LRR domains among these genes was negatively correlated with expression level after nematode infection. Thus, the WRKY transcription factor may possibly regulate LRR-containing genes associated with resistance to nematode infection. Our results may clarify physiological mechanisms of resistance to nematode infection and marker-assisted breeding in peanut.

## Additional files

Additional file 1: **Table S1.** Gene location, gene name, and domain type for LRR-containing genes from *Arachis duranensis (XLSX 59 kb)*

Additional file 2: **Figure S1.** The phylogenetic tree inferred using ATP binding amino acid sequences. Paralogs are indicated in blue. (TIF 310 kb)

Additional file 3: **Figure S2.** The phylogenetic tree inferred using F-box amino acid sequences. Paralogs are indicated in blue. (TIF 653 kb)

Additional file 4: **Figure S3.** The phylogenetic tree inferred using LRR receptor amino acid sequences. Paralogs are indicated in blue. (TIF 656 kb)

Additional file 5: **Figure S4.** The phylogenetic tree inferred using NBS–LRR amino acid sequences. Paralogs are indicated in blue. (TIF 617 kb)

Additional file 6: **Figure S5.** The phylogenetic tree inferred using protein kinase amino acid sequences. Paralogs are indicated in blue. (TIF 668 kb)

Additional file 7: **Figure S6.** The phylogenetic tree inferred using receptor-like kinase amino acid sequences. Paralogs are indicated in blue. (TIF 1634 kb)

Additional file 8: **Table S2.** Gene expression, gene location, gene name, and domain type among differentially expressed LRR-containing genes 3, 6, and 9 days after nematode infection. (XLSX 12 kb)

## Abbreviations

bHLH: basic/helix-loop-helix
ETI: Effector-triggered immunity
EXP: Expansin
FPKM: Fragments per kilobase of transcript per million mapped reads
GO: Gene ontology
HMM: Hidden Markov model
HSF: Heat shock transcription factor
JTT: Jones–Taylor–Thornton
*K*_a_: Nonsynonymous substitution ratio
*K*_a_/*K*_s_: Nonsynonymous to synonymous substitution ratio
*K*_s_: Synonymous substitution ratio
LOX: Lipoxygenase
LRR: Leucine-rich repeat
NBS–LRR: Nucleotide binding–leucine rich repeat
PAMP: Pathogen-associated molecular pattern
PRR: Pattern recognition receptor
PTI: Pattern-triggered immunity
WGCNA: Weighted gene co-expression network analysis
