# Supplementary figures and images for: Genome-wide identification of LRR-containing sequences and the response of these sequences to nematode infection in Arachis duranensis
Source: BMC Plant Biol. 2018 Nov 13;18:279. doi: 10.1186/s12870-018-1508-x (PMC6234637; doi:10.1186/s12870-018-1508-x)

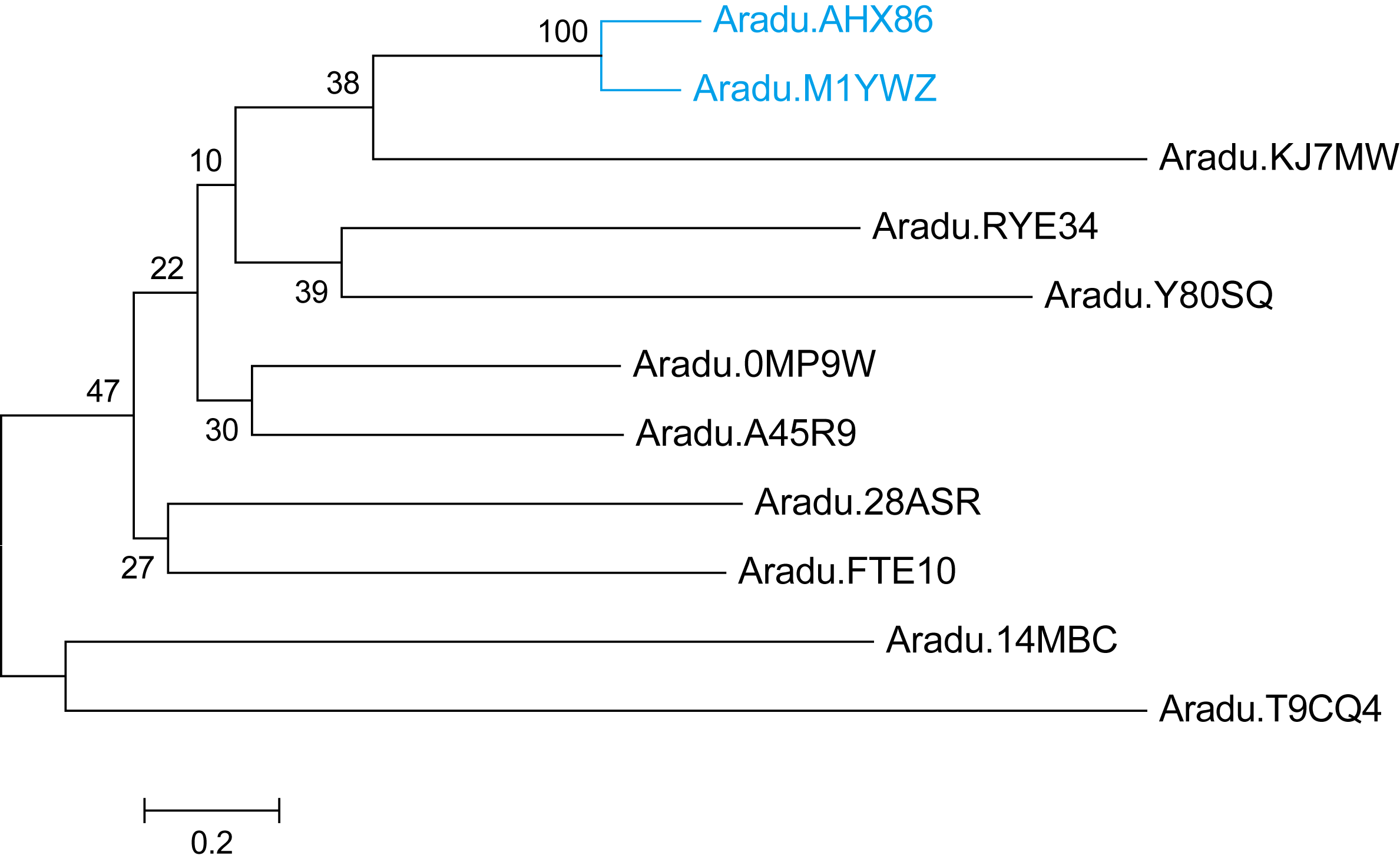

Supplement: Supplementary file 2 — Figure S1. The phylogenetic tree inferred using ATP binding amino acid sequences. Paralogs are indicated in blue. (TIF 310 kb) [file 12870_2018_1508_MOESM2_ESM.tif]

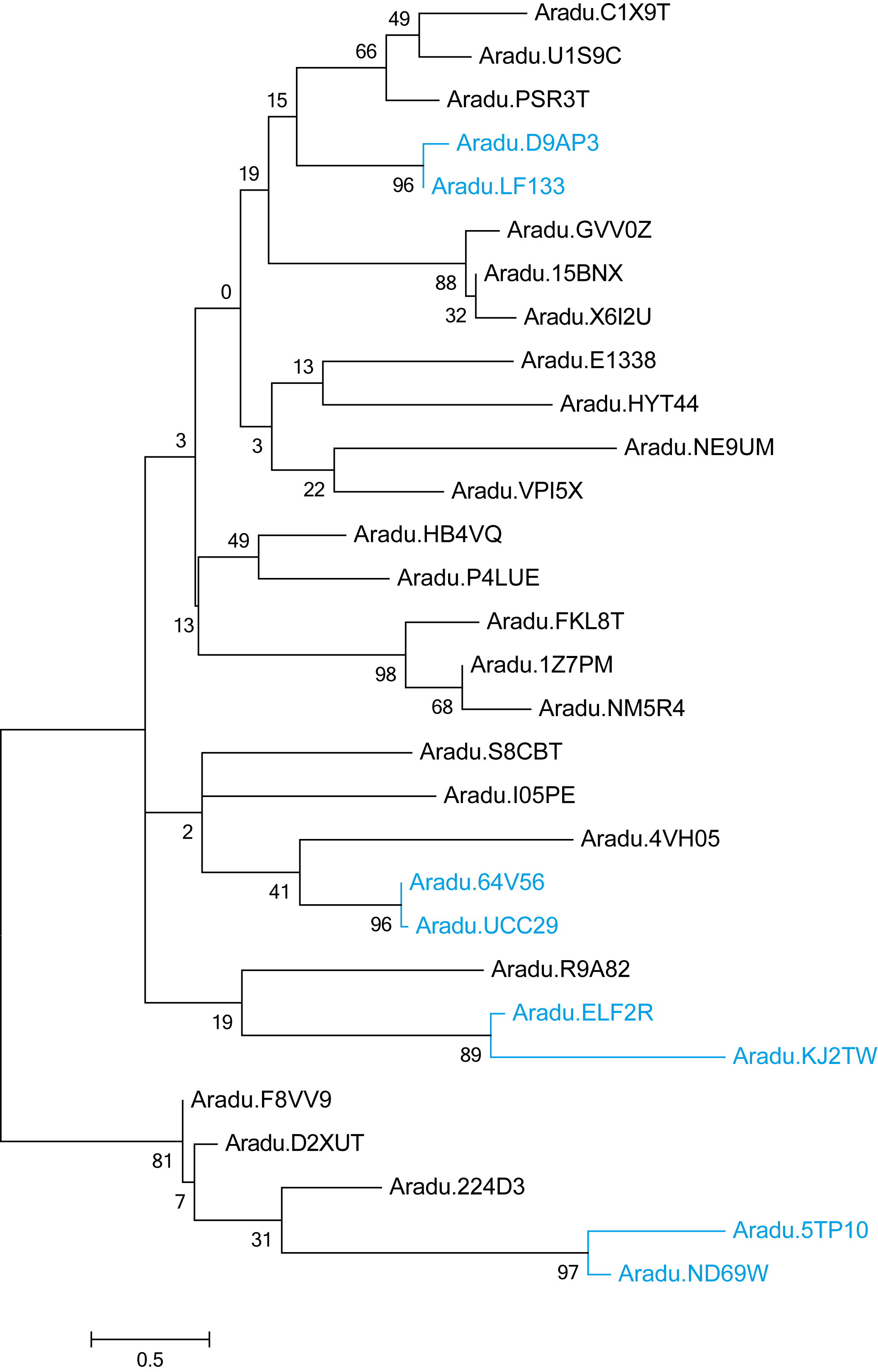

Supplement: Supplementary file 3 — Figure S2. The phylogenetic tree inferred using F-box amino acid sequences. Paralogs are indicated in blue. (TIF 653 kb) [file 12870_2018_1508_MOESM3_ESM.tif]

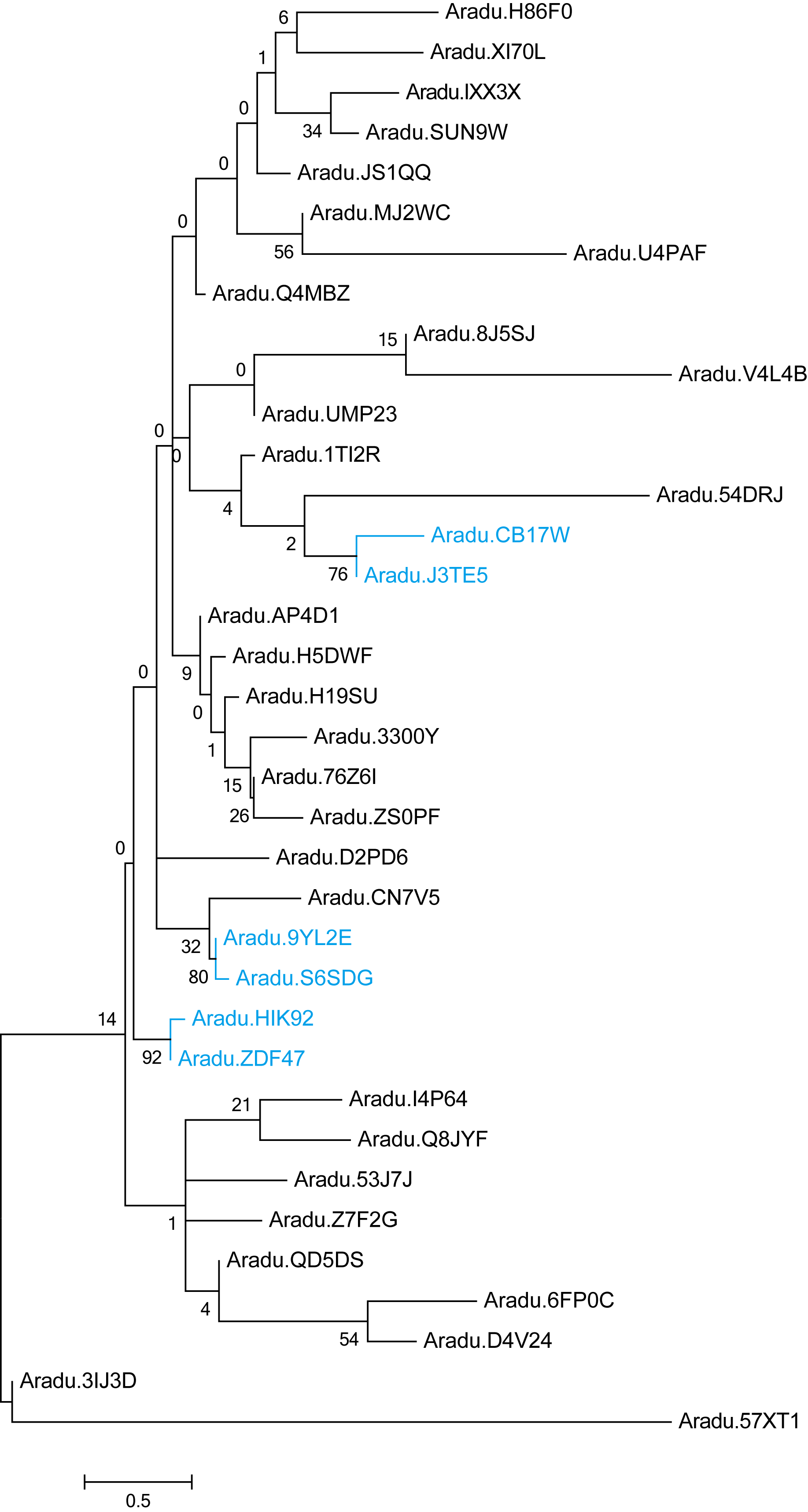

Supplement: Supplementary file 4 — Figure S3. The phylogenetic tree inferred using LRR receptor amino acid sequences. Paralogs are indicated in blue. (TIF 656 kb) [file 12870_2018_1508_MOESM4_ESM.tif]

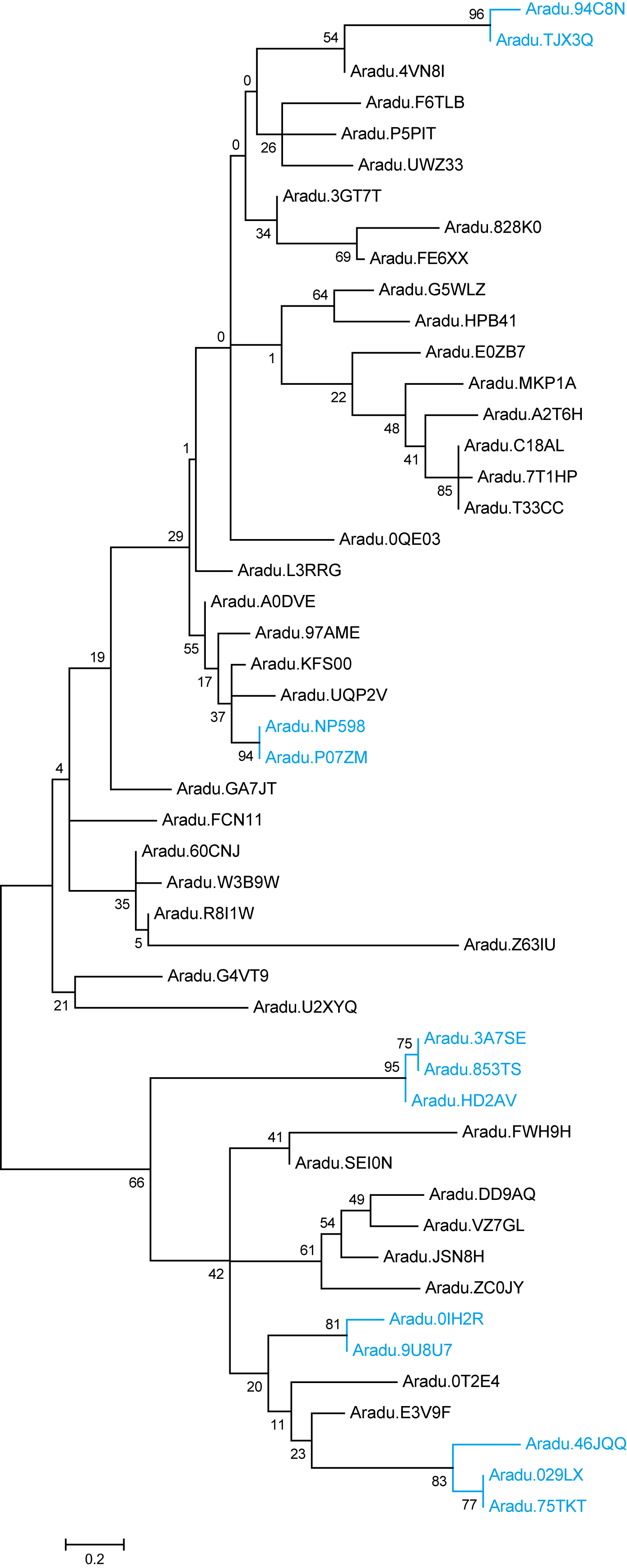

Supplement: Supplementary file 5 — Figure S4. The phylogenetic tree inferred using NBS–LRR amino acid sequences. Paralogs are indicated in blue. (TIF 617 kb) [file 12870_2018_1508_MOESM5_ESM.tif]

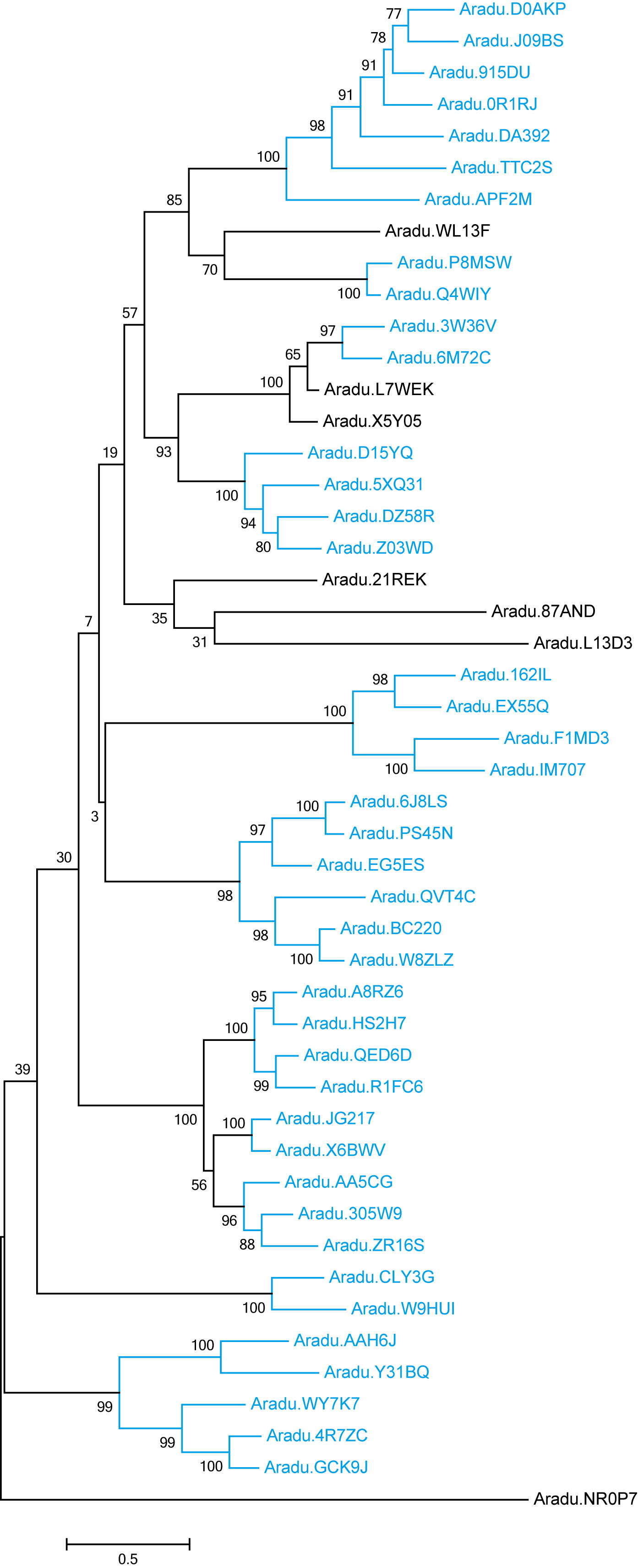

Supplement: Supplementary file 6 — Figure S5. The phylogenetic tree inferred using protein kinase amino acid sequences. Paralogs are indicated in blue. (TIF 668 kb) [file 12870_2018_1508_MOESM6_ESM.tif]

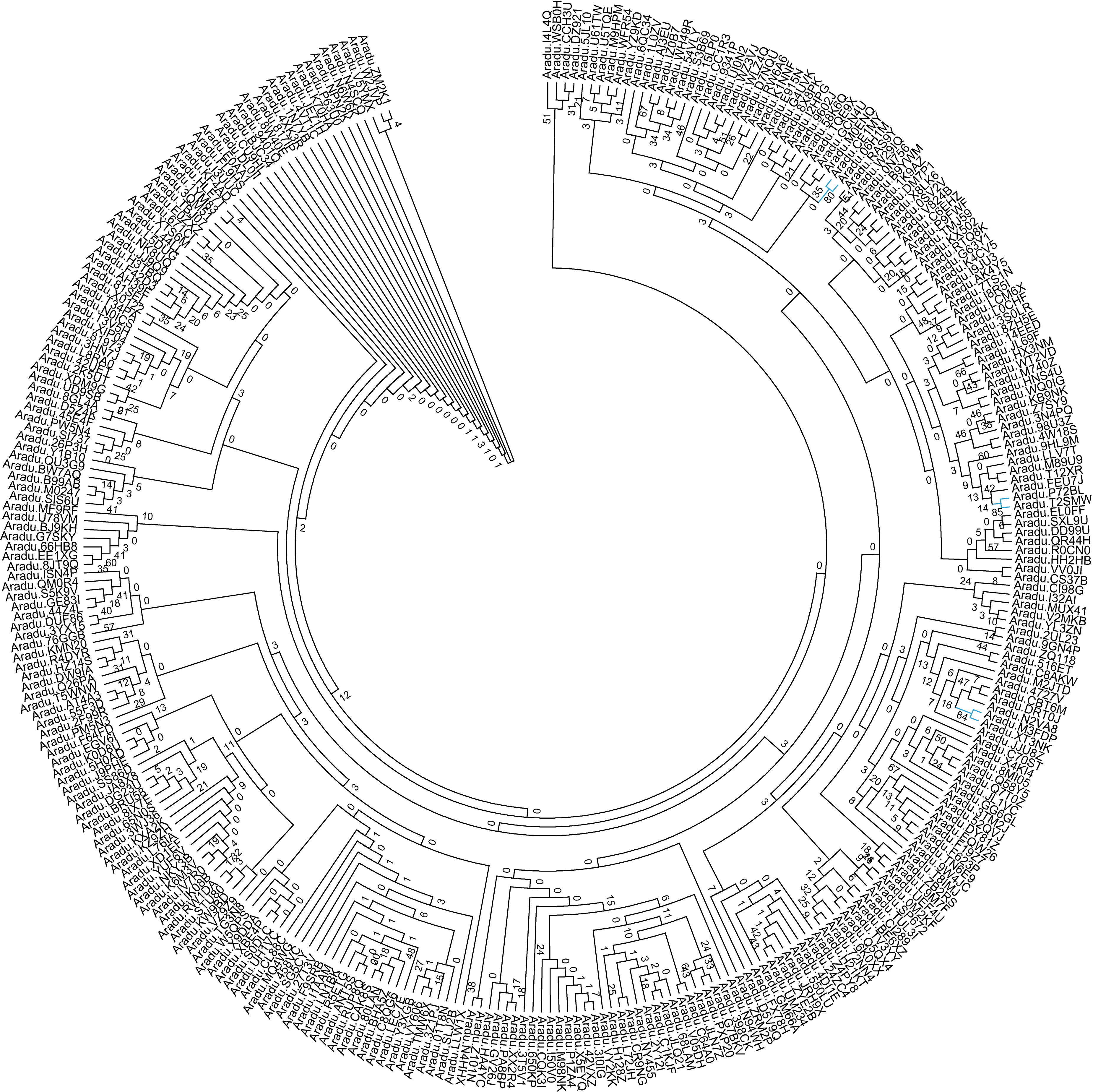

Supplement: Supplementary file 7 — Figure S6. The phylogenetic tree inferred using receptor-like kinase amino acid sequences. Paralogs are indicated in blue. (TIF 1634 kb) [file 12870_2018_1508_MOESM7_ESM.tif]
